# Effect of a single or two doses of an anti-GnRH vaccine on testicle morpho-functional characteristics in Nelore bulls

**DOI:** 10.1007/s11250-021-02600-x

**Published:** 2021-02-06

**Authors:** Emanuel M. Doroteu, Joao H. M. Viana, Jair A. Ferreira Junior, Juliana T. A. Macedo, Rodrigo A. Oliveira, Pedro M. O. Pedroso

**Affiliations:** 1 Universidade de Brasília, Brasília, DF 70636-200 Brazil; 2 Universidade de Brasília, Campus Universitário Darci Ribeiro, ICC, Ala Sul, Asa Norte, Brasília, DF 70297-400 Brazil; 3 Embrapa Recursos Genéticos e Biotecnologia, Brasília, DF 70770-190 Brazil

**Keywords:** Bopriva, Cattle, Immunocastration, Sperm

## Abstract

The aim of this study was to compare testicle morpho-functional characteristics in bulls undergoing a single or two immunizations against GnRH. Nelore (*Bos taurus indicus*) bulls were randomly allocated into three experimental groups: G1 (*n*=12), a single 400 μg dose of anti-GnRH vaccine on day 0; G2 (*n*=11), a first 400 μg dose of anti-GnRH vaccine on day 0 followed by a second (boost) dose 30 days later; and control group (CG, *n*=12), 1 mL saline 0.9% at day 0. Every 30 days, from day 0 until slaughter at day 90, the bulls were weighed and underwent testicular biometry, semen collection and analysis, and blood sample collection for testosterone measurement. Immediately after slaughter, the testicles were removed and transport at 15°C to the laboratory for histopathological analysis. There was a decrease in testicular height (*P*=0.0476), width (*P*=0.0021), and in scrotal circumference (*P*=0.0001), after either a single (G1) or two (G2) immunizations against GnRH. Both G1 and G2 had lower testosterone concentrations than CG from day 60 on (*P*<0.01), but in G2, it was also lower than in G1 at day 90 (*P*=0.0006). All sperm parameters were affected by active immunization against GnRH (*P*<0.05), and in G2, averages were lesser (*P*<0.05) than in G1 from day 60 on. No signs of seminiferous tubule degeneration were found in any sample from the CG, contrasting with 75.0% and 100.0% of the samples from G1 and G2, respectively. In summary, immunocastration affected testicle morpho-functional characteristics in bulls in a time- and dose-dependent way.

## Introduction

Castration of male cattle is a practice adopted worldwide by the beef industry. Besides preventing reproduction, the surgical removal of the testicles (orchiectomy) impairs the production of testosterone and thus reduces typical male aggressive behavior, attenuates stress, prevents accidents resulting from mounting acts, and facilitates cattle handling in confinement. Additionally, it may improve meat marble and tenderness (Marti et al. 2013) while reducing fights in the pre-slaughter period and subsequent downstream effects such as altered meat pH and color (Quaresma et al. 2017).

However, castration has a number of side effects. Depending on the castration methods, feed intake, feed conversion, and weight gain can be affected (Marti et al. 2013, 2015; Rodriguez et al. 2014). Surgically castrated steers undergo inflammation (Marti et al. 2017) and long wound healing periods (Mintline et al. 2014), causing extra expenses with labor and medication. Castration will also cause acute pain (Meléndez et al. 2017) and stress (Dockweiler et al. 2013). Moreover, there is an increasing public concern about animal welfare, and a negative perception on surgical castration when performed without pain control (Wolf et al. 2016; Lemos Teixeira et al. 2018).

Immunocastration has been proposed as an alternative for surgical castration. Synthetic GnRH conjugated with adjuvants can be used to induce the production of anti-GnRH antibodies and thus neutralize endogenous GnRH by active immunization (Thompson 2000). The effects of GnRH suppression by anti-GnRH vaccines include reduction in hypothalamic LH and FSH release, gonad development and size, spermatogenesis, and testosterone production (Jeffcoate et al. 1982; Monleón et al. 2020). Immunization against GnRH is also effective to suppress ovulation and estrous cycles in cows and heifers (Bishop et al. 1996; Balet et al. 2014).

Anti-GnRH vaccines are currently commercially available for immunocastration of cattle and pigs (Janett et al. 2012; Wicks et al. 2013) and are an animal welfare-friendly alternative to conventional castration (Marti et al. 2015). The most commonly recommended schedule of use is based on an initial immunization, followed by a boost dose of the antigen 30 days later (Janett et al. 2012) to ensure a greater response, both quantitatively (high concentration of antibodies) and qualitatively (speed of the response, type of antibody produced) (Jeffcoate et al. 1982; Adams 2005). However, the timing of the effects on morpho-functional characteristics of the testes has not yet been established for different vaccination schedules (Needham et al. 2019). Moreover, in most studies, the effects of immunization with a single dose of the vaccine were not evaluated. This could be a model to indirectly evaluate the relationship between antibody titers and the effects of immunization over time.

In the current study, we evaluated testicular, endocrine, and sperm characteristics over time in bulls undergoing a single or two active immunizations against GnRH. We hypothesized that changes in all endpoints would be time-dependent, with differences in magnitude according to the number of doses used for immunization.

## Materials and methods

### Animals and facilities

The experiment was conducted in a private beef farm, located in the municipality of Chapada Gaúcha (15° 18′ 20″ S; 45° 37′ 06″ W; 858 m altitude), northwest of Minas Gerais State, Brazil. Sound Nelore (*Bos taurus indicus*, *n*=35) bulls with an average 24 months age and 426.6±2.6 kg body weight were used. All bulls met the criteria recommended by the Brazilian College of Animal Reproduction (Colégio Brasileiro de Reprodução Animal 2013) for fertility, as confirmed by andrological examination: sperm motility ≥ 60%, vigor ≥ 3, swirling ≥ 3, minor defects ≤ 20%, major defects ≤ 10%, and percentage of normal cells ≥ 70%. The bulls were housed in a 50 ha *Panicum maximum* variety Mombasa pasture, with ad libitum access to water and salt, and received a diet formulated on the farm, containing sugar cane (76.2%), corn grain (18.6%), soybean meal (3.6%), mineral mix (1.0%), and urea (0.5%). The experiment started after a 15-day adaptation period. Bull handlings for biometric evaluations and semen collection were performed in a beef cattle squeeze chute. This study was approved by the Ethics in the Use of Animals Committee of the Universidade de Brasília (CEUA, Protocol #042/2020).

### Experimental design

The bulls were randomly assigned into three experimental groups: group 1 (G1, *n*=11), a single 400 μg dose of anti-GnRH vaccine (Bopriva, Zoetis, São Paulo, SP, Brazil) on day 0; group 2 (G2, *n*=12), a first 400 μg dose of anti-GnRH vaccine on day 0 followed by a second (boost) dose 30 days later; and control group (CG, *n*=12), 1 mL saline 0.9% at day 0. All treatments were injected SC in the left lateral region of the neck. Every 30 days, from day 0 until humane slaughter at day 90, the bulls were weighed and underwent testicular biometry, semen collection and analysis, and blood sample collection. Immediately after slaughter, the testicles were individually identified, removed, placed in plastic bags, and transported at 15°C to the Laboratory of Veterinary Pathology of the University of Brasília, for histopathological analysis. As an additional data, carcasses were evaluated for dressing percentage, and for fat coverage, according to the criteria used in a commercial slaughterhouse: absent, 0 mm; low, 1 to 3 mm; medium, 4 to 6 mm; uniform, 7 to 10 mm; and excessive, over 10 mm.

### Testicular biometry and semen quality analysis

During biometric evaluations, each testicle was scanned by ultrasonography, using a 7.5 MHz liner probe (Mindray DP 2200, Mindray do Brasil, Monções, SP, Brazil), and testicle height and width were measured in the longitudinal and transversal images of the testicular parenchyma, respectively. Ultrasound settings (focus, gains, brightness, and contrast) were standardized for all examinations. The circumference of the scrotum was measured using a specific tape for scrotal perimeter (Biocom, Uberaba, MG, Brazil).

Semen collection was performed by electroejaculation. A sample of 20 μL of fresh semen was evaluated under optical microscopy (×200 magnification) for mass movement (swirling), motility, and vigor. Another 20 μL aliquot was stained with 20 μL of Bengal Rose dye for morphological analysis of the sperm cells (×1000 magnification). Sperm concentration was calculated using a spermiodensimeter (Karras, Minitub, GmbH, Tiefenbach, Germany), as previously described (Vianna et al. 2007).

### Serum testosterone concentration

Blood samples were collected from the jugular vein using 21G needles and vacuolized tubes (Vacutainer Systems, Becton Dickinson, Sao Paulo, SP, Brazil). Serum was obtained by blood sample centrifugation for 5 min at 2078×*g*, and stored at −20°C for further analysis. Testosterone concentration was measured by electro-chemiluminescence immunoassay (ECL), using an Elecsys analyzer device (Indianapolis, IN, USA; test sensitivity = 3 ng/dL). The intra- and inter-assay coefficients of variance were 7.3% and 3.4%, respectively.

### Anatomopathological analysis

Testicles were first sectioned in 1.0- to 2.0-cm-thick fragments. Samples pieces of the head, body, and tail of the epididymis, as well as of the testicular parenchyma, were fixed in 10% buffered formalin solution for 48 h. The fragments were then routinely processed for histology, embedded in paraffin, cut into 4 μ slices, and stained with hematoxylin and eosin.

The presence of vacuolization, nuclear fragments, or pyknosis, as well as a reduction in the number of tubular lining cells in the seminiferous epithelium, were considered signs of degeneration of the seminiferous tubules. We evaluated 10 fields of each slide, and the degeneration was graded as follows: absent (0), when no changes were observed; mild (1), when signs of degeneration was observed in ≤ 50% of the analyzed fields; moderate (2), when 50–75% of the fields presented degenerative changes; severe (3), when few seminiferous tubular lining cells were present and > 75% of the seminiferous tubules were affected; and complete (4), when seminiferous tubular epithelium is mostly degraded and no sperm cell is recognizable (Cavalieri et al. 2015).

### Statistical analysis

For each bull, averages of the data from the left and right testicles for linear measurements, as well as for histopathology, were used. The data of testicular biometry, sperm analysis, and testosterone concentration were initially tested for normality using the Shapiro-Wilk test. Analyses considered the main effects of treatment (CG, G1, and G2), time (days), and their interactions. The SAS GLIMMIX procedure with a REPEATED statement was used to account for auto-correlation between sequential measures (SAS University Edition, SAS Institute Inc., Cary, NC, USA). The model was adjusted for the type of distribution of each endpoint. Differences between means were compared using the Tukey test. Data of weight and carcass traits was analyzed using the GLIMMIX procedure of the SAS. The grades of seminiferous tubule degeneration were compared among groups using the Kruskal-Wallis non-parametric test. Associations between the degree of degeneration of the seminiferous tubules and other testicle parameters at day 90 were calculated using the Pearson’s correlation method. Results are presented as mean ± S.E.M. A *P* value lower than 0.05 indicated statistical significance.

## Results

**Fig. 1:**
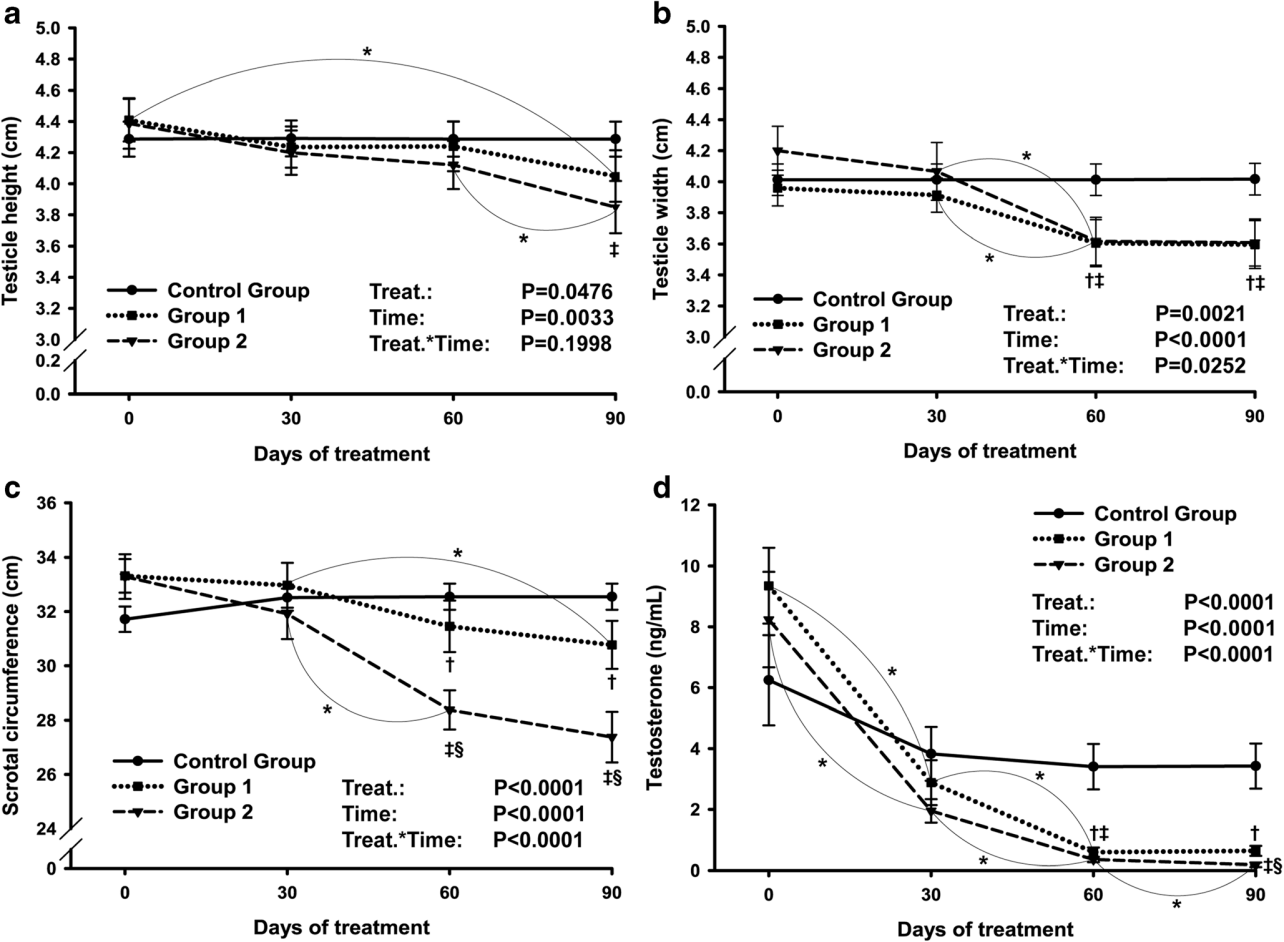
**a**–**d** Testicular and endocrine endpoints in Nelore bulls immunized against GnRH once (group 1), twice (group 2), and controls (control group). **a** Testicle height, **b** testicle width, **c** scrotal circumference, and **d** serum testosterone concentration. *Difference (*P*<0.05) over time for the same group; †G1 vs CG (*P*<0.05); ‡G2 vs CG (*P*<0.05); §G1 vs G2 (*P*<0.05)

The data of testicle height and width, scrotum circumference, and testosterone concentrations are shown in Fig. 1a to d. All these endpoints decreased over time after either a single (G1) or two (G2) active immunizations against GnRH (*P*<0.01). Testicular width (Fig. 1b), scrotal circumference (Fig. 1c), and testosterone concentration (Fig. 1d) were lesser (*P*<0.05) in both G1 and G2 from day 60 on, compared with control group (CG). The testicular height (Fig. 1a) in G2, but not in G1, differed for CG, and only at day 90 (*P*<0.01). The boost immunization at day 30 resulted in lesser (*P*<0.001) scrotal circumference in G2, compared with G1, at days 60 and 90 (Fig. 1c), and in lower testosterone concentration (Fig. 1d) at day 90.

**Fig. 2:**
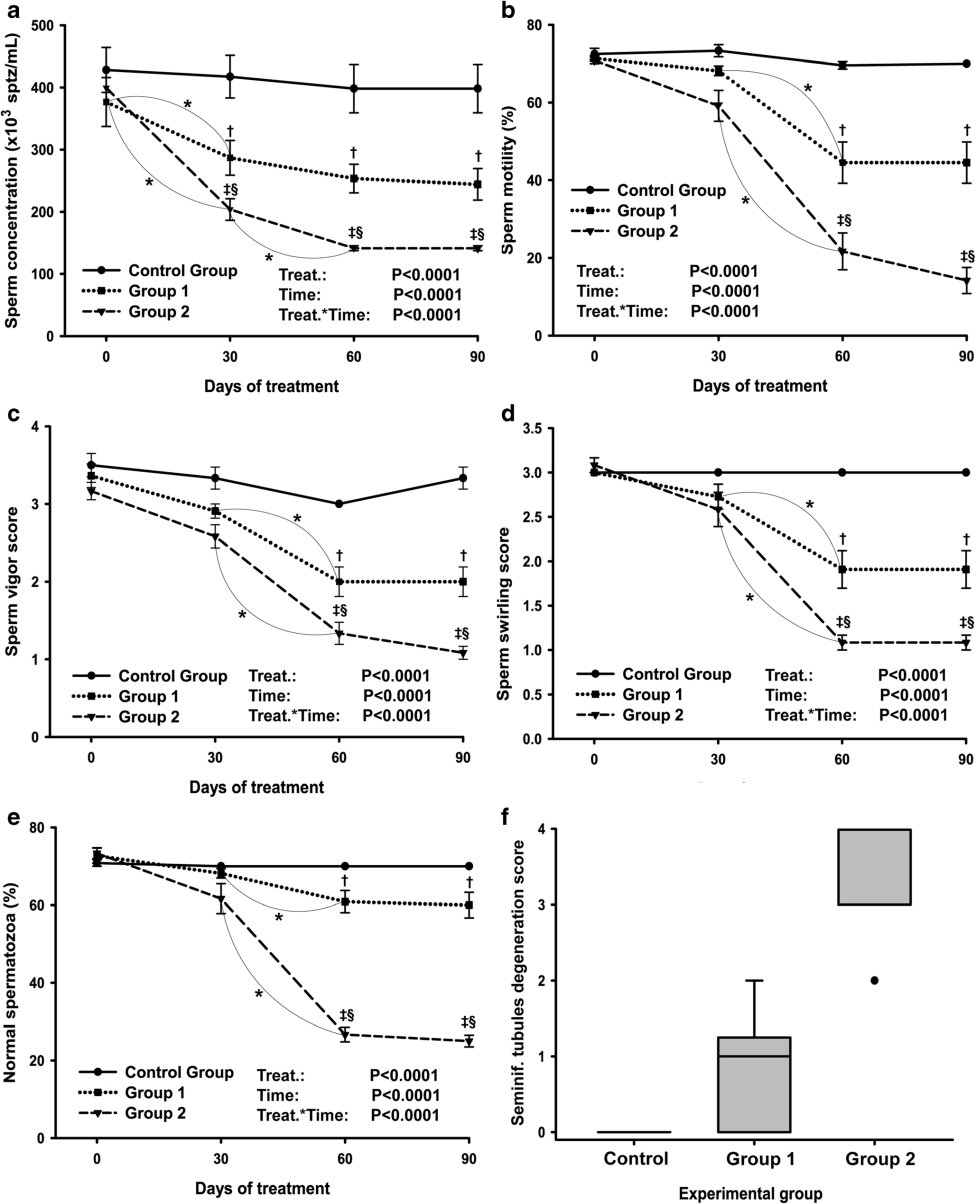
**a**–**f** Sperm endpoints and seminiferous tubule degeneration in Nelore bulls immunized against GnRH once (group 1), twice (group 2), and controls (control group). **a** Sperm concentration, **b** sperm motility, **c** sperm vigor score (1 to 5 scale), **d** sperm swirling score (1 to 5 scale), **e** normal spermatozoa, and **f** boxplot of seminiferous tubule degeneration degree. *Difference (*P*<0.05) over time for the same group; †G1 vs CG (*P*<0.05); ‡G2 vs CG (*P*<0.05); §G1 vs G2 (*P*<0.05)

Endpoints of sperm analysis and histological testicular degeneration are shown in Fig. 2. All sperm parameters were affected by active immunization against GnRH and decreased over time (Fig. 2a to e; *P*<0.0001). Sperm concentration (Fig. 2a) in G2 was lesser than in G1, and both were lesser than CG from day 30 (*P*<0.01), while for sperm motility (Fig. 2b), vigor (Fig. 2c), swirling (Fig. 2d), and proportion of normal spermatozoa (Fig. 2e), such differences were observed from day 60 on (*P*<0.0001). In G2, two bulls presented complete azoospermia.

**Fig. 3:**
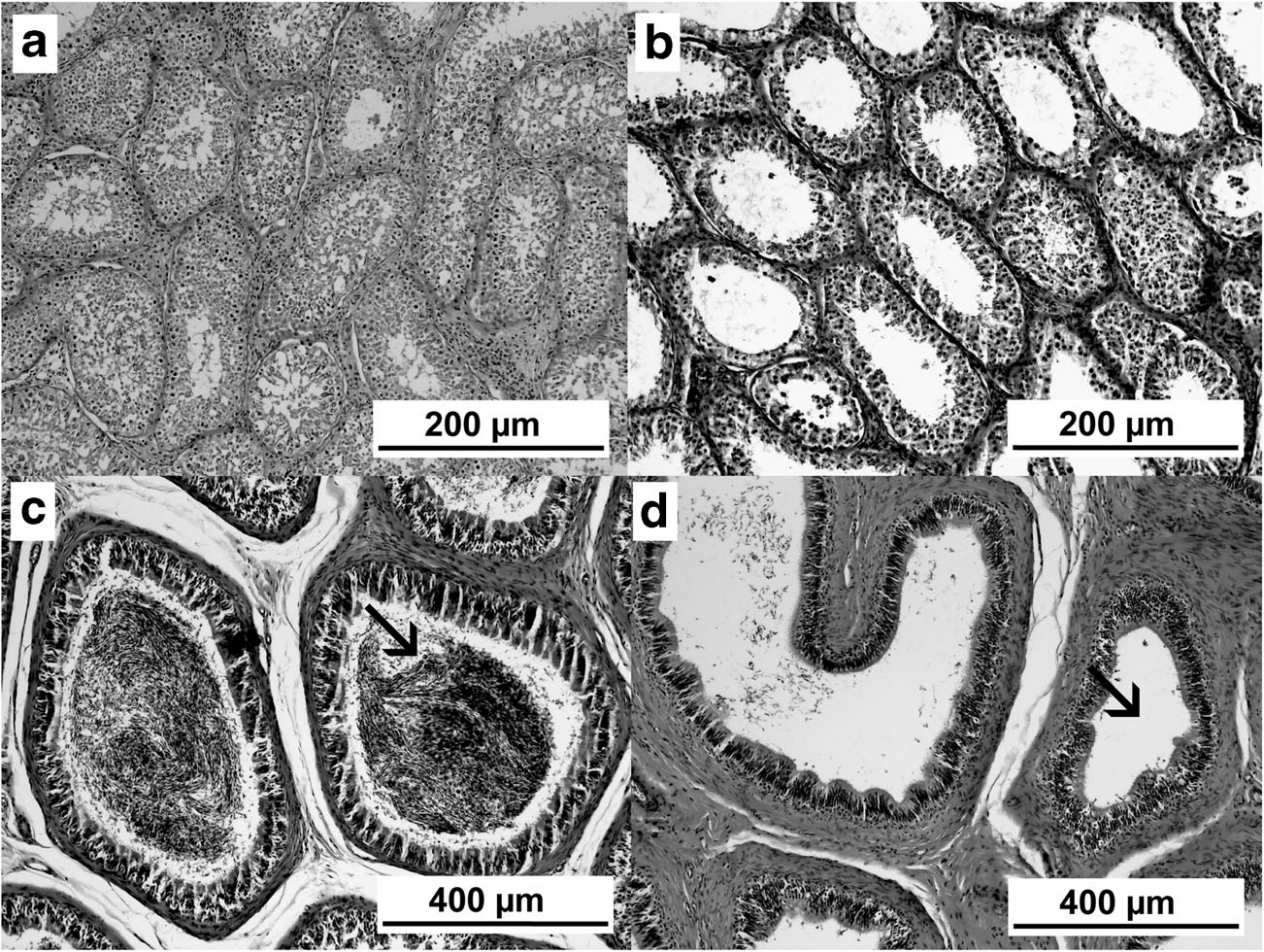
**a**–**d** Representative images from histological sections of the testicles (**a**–**b**, ×100 magnification) and epididymis (**c**–**d**, ×400 magnification) from Nelore bulls immunized or not against GnRH. (**a**) Normal seminiferous tubules. (**b**) Severe (grade 3) degeneration at the seminiferous tubules. (**c**) Normal epididymal ducts from epididymis head, with lumen occupied with sperm (arrow). (**d**) Epididymal ducts showing an atrophied epithelium and no detectable sperm (arrow)

No signs of seminiferous tubule degeneration were found after histopathological evaluation of the testicle samples from the CG. In contrast, degeneration was present in 75.0% and 100.0% of the samples from G1 and G2, respectively. The severity of degeneration was also greater according to the number of immunizations (grade 0.9±0.1 in G1 vs grade 3.1±0.2 in G2, *P*<0.01). The boxplot distribution of the degree of degeneration in each group is shown in Fig. 2f, and representative images are shown in Fig. 3a to d. In immunized bulls, the germinative epithelium of the seminiferous tubules underwent changes in the number of spermatogonia, spermatocytes, and spermatozoa, and multinucleated giant cells were observed. The atrophy of the seminiferous epithelium (Fig. 3b) and a reduction in the size and thickness of the epididymal ducts (Fig. 3d) coincided with areas where reduced or lack of spermatogenesis was observed. The correlations between the degree of degeneration of the seminiferous tubules and other testicle parameters at day 90 are shown in Table 1.

**Table 1:** Associations between the degree of degeneration of seminiferous tubules, as scored after histopathology analysis, and other morphological and functional testicle parameters immediately before slaughter

Endpoint	*R*	*P* value
Scrotal circumference	−0.56	0.0004
Testicle height	−0.28	0.1014
Testicle width	−0.23	0.1785
Serum testosterone	−0.52	0.0012
Sperm concentration	−0.69	<0.0001
Sperm motility	−0.82	<0.0001
Sperm vigor	−0.79	<0.0001
Sperm swirling	−0.79	<0.0001
Percentage of normal SPTZ	−0.87	<0.0001

*R*= Pearson’s coefficients of correlation

Albeit a difference in final weight (at day 90) between CG and G1, there was no (*P*>0.05) difference in total or in average daily weight gain among groups (Table 2). However, the dressing percentage was greater in G2 than in G1 and in CG (*P*=0.0102). All bulls presented a low (1 to 3 mm) subcutaneous fat coverage.

**Table 2:** Weight and carcass traits in Nelore bulls immunized against GnRH once (group 1), twice (group 2), and controls (control group)

Endpoint	Control group	Group 1	Group 2	*P* value
Initial weight (kg)	433.8±5.8	418.6±2.4	426.7±3.1	0.0513
Final weight (kg)	557.9±8.3^a^	528.6±5.0^b^	539.2±6.0^ab^	0.0141
Weight gain (kg)	124.2±5.0	110.0±4.5	112.5±5.4	0.1214
ADG (kg)	1.4±0.1	1.2±0.0	1.3±0.1	0.1155
Dressing percentage	50.0±0.1^b^	50.0±0.1^b^	50.3±0.1^a^	0.0102

*ADG* average daily gain

^a,b^Means with different superscripts, in the same row, differ (*P*<0.05)

## Discussion

In the current study, we evaluated the effects of a single or two active immunizations against GnRH on reproductive parameters of bulls. We confirmed the hypothesis that the various effects of GnRH suppression on the morpho-physiology and function of the testicles occur progressively after immunization. The novelty of this study was to evaluate the use of a single dose of the anti-GnRH vaccine (G1), which resulted in overall milder effects on scrotal circumference, sperm production and quality, and in testosterone levels, compared with the group receiving a boost 30 days after the first dose (G2).

There was no difference among groups on day 0 for any of the parameters studied, demonstrating that there was no bias in the distribution of the bulls among experimental groups. Similarly, no significant change occurred over time in any endpoint at the control group, while a time effect was observed consistently in G1 and G2, with significant reductions in averages being observed throughout the evaluation period for all morpho-functional endpoints studied. Interestingly, in our study a single immunization against GnRH significantly affected all the testicular morpho-functional parameters, although in a lesser degree than in the group receiving a boost dose of the vaccine. This result differs from previous reports in which significant differences in testicular characteristics were only detected after the second treatment (Cook et al. 2000). Actually, detectable antibody titers are present after the first immunization (Amatayakul-Chantler et al. 2012; Balet et al. 2014), even in the absence of immunological memory driven by a previous exposition to the antigen. We can speculate that, in the current study, such titers were able to cause a consistent reduction in circulating FSH and LH, as the effects on testis function persisted up to the 12th week.

As expected, the changes observed in testicle functional endpoints were of greater magnitude than those observed in morphology (overall −72.4% vs −12.1%, respectively). The Sertoli cells are dependent on FSH support (Berndston and Desjardins 1974), and the decreases in FSH release by the hypothalamus subsequent to GnRH immunization have direct detrimental effects on Sertoli cells function and survival (Monleón et al. 2020). As result, spermatogenesis and spermiogenesis are compromised, with substantial reduction in sperm concentration and quality. On the other hand, differences in testicular biometry are driven mostly by the organ atresia subsequent to the degeneration of the seminiferous tubules, affecting testicle size by cellular mechanisms that will lead to cell death and tissue loss. In fact, the degree of degeneration of the seminiferous tubules and the sperm characteristics just before slaughter were strongly correlated (*R* ranging from −0.69 to −0.87; *P*<0.0001), while linear testicle measurements were not (*R*= 0.28 and 0.23; *P*>0.10). Thus, a mild degree of degeneration will be sufficient to cause a significant drop in sperm quality, but may be not enough to cause a proportional reduction in testicular size.

In the current experiment, no difference was observed at any time-point in testicle height or width between G1 and G2, contrasting with the differences observed in scrotal circumference at days 60 and 90. The changes in scrotal circumference were more consistent with those observed for sperm endpoints, in which the effects of immunization on G2 were of greater magnitude than in G1. In this regard, scrotal circumference was more sensitive to detect differences among groups compared to single, linear measurements of height or width. The measurement of scrotal circumference reflects the transversal area of the testicles, compensating possible variations in testicle shape among animals that could bias the width. On the other hand, testicle height was only different between CG and G2 and at day 90, as observed in previous studies (D'Occhio et al. 2001; Xu et al. 2018).

The concurrent lack of LH support for Leydig cells resulted in a drop of testosterone production after treatment, and serum testosterone in both G1 and G2 differed from control from day 60 on. However, in G1 testosterone concentrations stabilized after day 60, while in G2 they decreased until day 90, reaching values close to zero and differing from G1. In fact, a previous study reported testosterone concentrations after the second immunization with Bopriva similar to those observed in surgically castrated bulls (Amatayakul-Chantler et al. 2013). This observation suggests that anti-GnRH titers were already high enough in G1 to affect hypophysis LH secretion with a single dose (Needham et al. 2019), although probably much lower than the titers normally found after the boost (Amatayakul-Chantler et al. 2012). As the main goal of immunocastration in the beef industry is to reduce aggressive behavior, rather than being an anticonception strategy, the use of a single dose of the vaccine could be an alternative to reduce testosterone concentrations for short periods (up to 90 days) with lower costs. However, on must take in account that the production of antibodies after immunization against GnRH is highly variable among individuals (Balet et al. 2014), and other studies did not observe changes in testosterone concentrations before the second injection of the vaccine (Janett et al. 2012; Marti et al. 2015). In the perspective of the commercial use of the vaccine, such potential variations in results are not desirable and may eventually overcome the economic advantages of using a single dose.

The dramatic drop in testosterone concentrations in G2 was likely to impact somatic development, due to the lack of its anabolic effects. As an additional evidence of the changes in testicular function, we evaluated body weight gain throughout the experiment, as well as the dressing percentage and subcutaneous fat deposition at slaughter. Despite of a lower final body weight in G1, compared with the CG, there was no difference among groups in total or in average daily weight gain. Previous studies reported that the lack of the anabolic effects of testosterone in castrated steers may affect the average weight gain (Cook et al. 2000). In the current experiment, however, a significant difference in testosterone serum concentrations between control and treated groups only occurred at days 60 and 90 and could speculate if the lack of differences in weight gain and in subcutaneous fat coverage could reflect the relatively short period from immunization to slaughter, as long-term effects could not be evaluated. On the other hand, the dressing percentage was greater in the group receiving two immunization vaccines (G2), as previously reported (Amatayakul-Chantler et al. 2013). Contrasting results (differences in weight gain, but not in carcass traits) were found when three doses of anti-GnRH vaccine were used; however, bulls were evaluated for a longer period (D'Occhio et al. 2001). In this regard, the potential effects of different immunocastration schedules on the performance and meat traits of cattle may be affected by the interactions with genetic background (Monteiro et al. 2014), finishing diet (Prado et al. 2015), and interval from vaccination to slaughter (Needham et al. 2019).

In summary, immunocastration affects testicle morphology, testosterone production, and sperm characteristics, in a time- and dose-dependent way. A single dose of an anti-GnRH vaccine affects all morphological and functional parameters but cause milder effects than the conventional schedule of two doses. Changes in morphological and functional characteristics in bulls receiving either one or two immunizations are observed at similar time-points, but with different intensity, which is likely related to the differences in antibodies titers.
